# Habitat-based radiomics enhances the ability to predict lymphovascular space invasion in cervical cancer: a multi-center study

**DOI:** 10.3389/fonc.2023.1252074

**Published:** 2023-10-26

**Authors:** Shuxing Wang, Xiaowen Liu, Yu Wu, Changsi Jiang, Yan Luo, Xue Tang, Rui Wang, Xiaochun Zhang, Jingshan Gong

**Affiliations:** ^1^ The Second Clinical Medical College, Jinan University, Shenzhen, China; ^2^ Department of Radiology, Guangzhou Women and Children’s Medical Center, Guangzhou, China; ^3^ Department of Radiology, Shenzhen People’s Hospital (The Second Clinical Medical College of Jinan University, The First Affiliated Hospital of Southern University of Science and Technology), Shenzhen, China

**Keywords:** cervical cancer, LVSI, radiomics, habitat, machine learning

## Abstract

**Introduction:**

Lymphovascular space invasion (LVSI) is associated with lymph node metastasis and poor prognosis in cervical cancer. In this study, we investigated the potential of radiomics, derived from magnetic resonance (MR) images using habitat analysis, as a non-invasive surrogate biomarker for predicting LVSI in cervical cancer.

**Methods:**

This retrospective study included 300 patients with cervical cancer who underwent surgical treatment at two centres (centre 1 = 198 and centre 2 = 102). Using the k-means clustering method, contrast-enhanced T1-weighted imaging (CE-T1WI) images were segmented based on voxel and entropy values, creating sub-regions within the volume ofinterest. Radiomics features were extracted from these sub-regions. Pearson correlation coefficient and least absolute shrinkage and selection operator LASSO) regression methods were used to select features associated with LVSI in cervical cancer. Support vector machine (SVM) model was developed based on the radiomics features extracted from each sub-region in the training cohort.

**Results:**

The voxels and entropy values of the CE-T1WI images were clustered into three sub-regions. In the training cohort, the AUCs of the SVM models based on radiomics features derived from the whole tumour, habitat 1, habitat 2, and habitat 3 models were 0.805 (95% confidence interval [CI]: 0.745–0.864), 0.873(95% CI: 0.824–0.922), 0.869 (95% CI: 0.821–0.917), and 0.870 (95% CI: 0.821–0.920), respectively. Compared with whole tumour model, the predictive performances of habitat 3 model was the highest in the external test cohort (0.780 [95% CI: 0.692–0.869]).

**Conclusions:**

The radiomics model based on the tumour sub-regional habitat demonstrated superior predictive performance for an LVSI in cervical cancer than that of radiomics model derived from the whole tumour.

## 1 Introduction

Cervical cancer is one of the most prevalent gynaecological malignancies worldwide, ranking fourth in cancer incidence among women (1). In 2020, approximately 110,000 new cases of cervical cancer were diagnosed in China alone, representing 18% of the new cases of cervical cancer worldwide (2). In some developing countries, the prevalence and mortality rates of cervical cancer surpasses those of breast cancer (3, 4). In cervical cancer, lymphovascular space invasion (LVSI), the infiltration of tumour cells into the blood and lymphatic vessels, is closely associated with lymph node metastasis and serves as an independent risk factor for prognosis (5–7). According to the 2018 International Federation of Gynecology and Obstetrics (FIGO) staging and treatment guidelines, the treatment decision for patients with stage IA1 cervical cancer should take into account the LVSI status. Patients with LVSI-positive lesions should undergo adjuvant chemoradiotherapy or additional radical resection and lymph node dissection surgery to suppress the spread of lymph node micrometastases and improve prognosis (8). Therefore, determining LVSI status is important for making treatment decision, especially in women of childbearing age who wish to preserve fertility.

Considering the high heterogeneity of the malignancies, tumours exhibit diverse microenvironments and microstructures (9–11). Radiomics, which involves extracting numerous features from medical images to classify diseases using machine-learning techniques, offers the potential to deliver personalised medicine in an no-invasive manner. Traditional radiomic analysis typically focuses on the whole tumour and overlooks the sub-regional phenotypic variations within the tumour (12). Recently, a new approach called habitat, which divides tumours into sub-regions by identifying grayscale voxels with comparable imaging characteristics (12, 13), has shown the potential in improving the ability to distinguish between tumour heterogeneity (14–16). In this study, we intended to extract radiomic signatures from different sub-regions of cervical cancer using contrast-enhanced T1-weighted imaging (CE-TIWI) with habitat analysis to decode the LVSI status, thereby facilitating personalised therapeutic decision making.

## 2 Materials and methods

This study was approved by two medical ethics committees that conducted ethical reviews and waived the requirement for obtaining patient consent.

### 2.1 Patient population

We recruited 300 patients with pathologically confirmed cervical cancer, who underwent pelvicmagnetic resonance (MR) imaging within 1 month before surgery and without any anti-tumour therapy before MR. Among them, 198 patients from centre 1 constituted the training cohort, whereas the remaining 102 from centre 2 constituted the external test cohort. We collected and organised two distinct datasets of MRI images from female patients diagnosed with cervical cancer using a picture archiving and communication system. The training cohort comprised 104 LVSI-positive and 94 LVSI-negative patients and the external test cohort comprised 54 LVSI-positive and 48 LVSI-negative patients. We retrospectively analysed clinical data and laboratory indicators, including age, maximum tumour diameter, histological classification, degree of cellular differentiation, FIGO stage, CA125 and CA199 levels, squamous cell carcinoma antigen, and human papillomavirus infection status. The inclusion criteria for the study population were as follows: 1) patients who underwent pelvic MRI before surgery and 2) LVSI confirmed by postoperative pathological examination. The exclusion criteria were as follows: 1) pregnant women; 2) those who underwent cervical conization or loop electrosurgical excision; 3) those who had a history of radiotherapy or chemotherapy before the MRI examination; and 4) those with blurry diagnostic images.

### 2.2 MRI protocols

**Figure 1:**
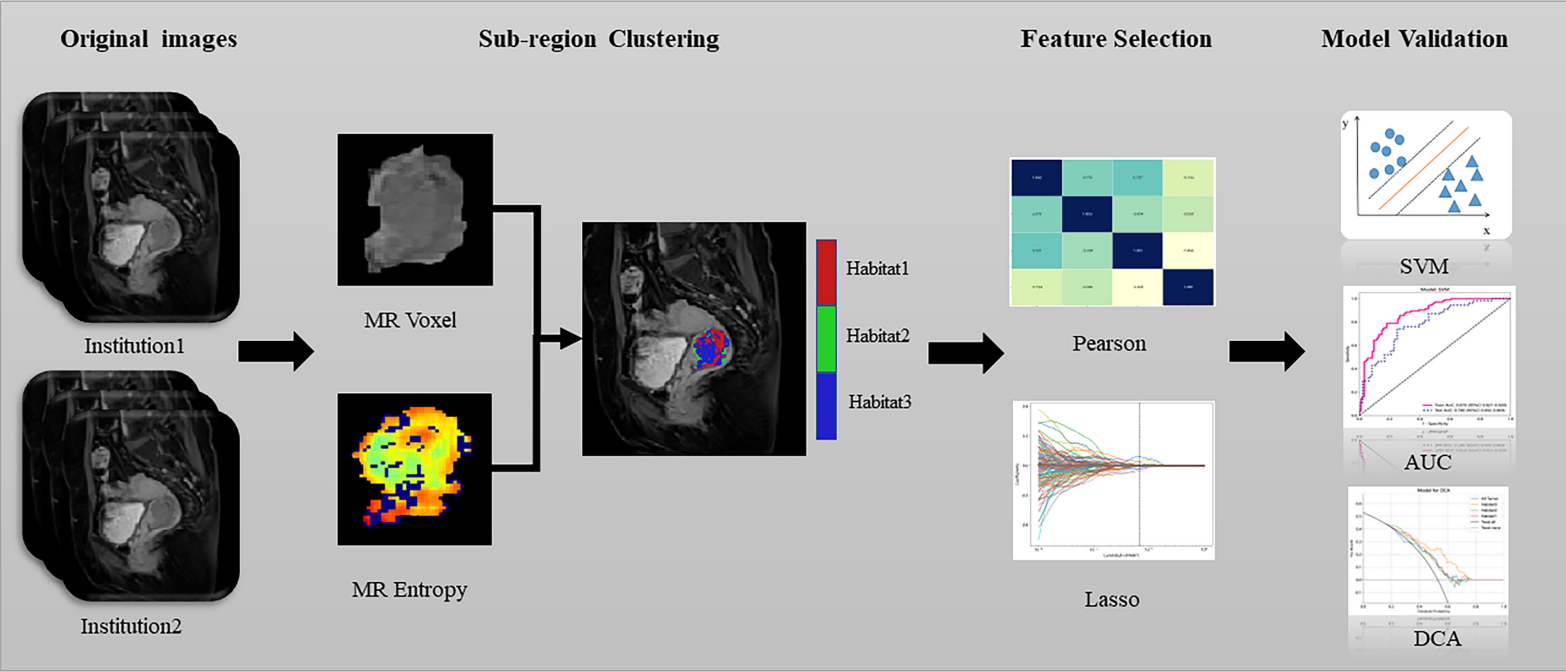
Flowchart showing the habitat analysis process.

The scanning protocol and parameters are included in the **Supplementary Material**. The CE-TIWI images were downloaded from the picture archiving and communication system and transferred to a personal computer. Two radiologists, each with more than 5 years of experience in pelvic diagnosis, segmented the tumours layer-by-layer on the CE-TIWI images using the open-source software ITK-SNAP (version 3.6, www.itk-snap.org) to obtain the volume of interest (VOI) with the aid of diffusion weighted image (DWI). After 1 week, 30 sets of CE-TIWI images were randomly selected, and the outlining process was repeated. Features with intraclass correlation coefficients (ICC) value of<0.75 were retained for screening. Any differences in the outlining process were resolved by a radiologist with over 15 years of experience. The two radiologists were blinded to the patients’ pathological diagnoses during the outlining process. A flowchart illustrating this process is presented in **Figure 1**.

### 2.3 VOI delineation and sub-region clustering

Habitat utilises voxel and entropy values derived from MR images to cluster VOIs into sub-regions (17–19). The voxel counts for each tumour VOI were determined using a traditional method, whereas the entropy values were computed for each layer of the MR images using the following formula:


$$V_{voxel}=\;\sum_{k=1}^{N_{v}}V_{k}$$

$$entropy\;=\;-\sum_{i=1}^{N_{g}}p\left(i\right)\log_{2}\left(p\left(i\right)+\epsilon \right)$$

**Figure 2:**
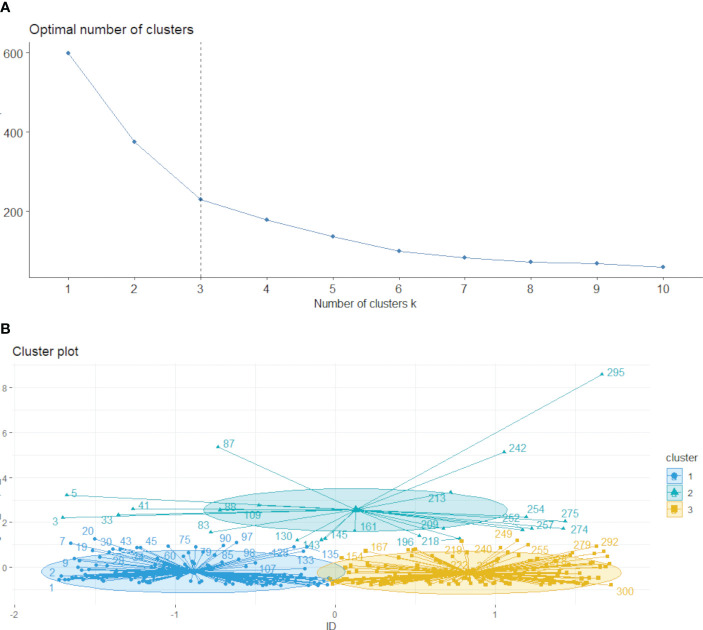
Based on the area change under the conditional density function curve. We observed that clustering separation was optimal at a k value of 3 **(A, B)**. This value corresponded to a sharp decrease in the area change under the receiver operating characteristic curve, which suggested that after this k value, further improvements in separability were negligible.

The k-means method was employed to cluster the VOI regions at the patient level, forming multiple habitats, and the distance correlation between samples was calculated using the Euclidean distance (voxel values and entropy values). The number of habitats was tested from 2 to 10, and the optimal k-value was selected using the Consensus Cluster Plus method, which evaluated the consistency of clustering features by resampling multiple voxels in the cluster 1000 times in 80% of the samples to select the k-value corresponding to a well-separated and stable cluster. The optimal k-value served as the criterion for selecting the optimal number of clusters at the patient population level (**Figure 2**). The optimal k-value was found to be 3. Using the OnekeyAI platform, we imported each patient’s VOI into the platform’s components and classified the cervical cancer tumours into three classes named habitat 1, habitat 2, and habitat 3.

### 2.4 Feature selection and model development

To account for differences in imaging features caused by variations in the reconstruction layer thickness and pixel size, the images were resampled to 1×1×3 m^3 and normalized to a grayscale range of 0–255. Features were independently extracted from each of the four habitats, habitat 1, habitat 2, habitat 3 and the whole tumour using the PyRadiomics program package (20), which adheres to the imaging biomarker standardization initiative (21). Before the feature extraction, two filters, wavelet and log-sigma, were implemented to enhance the process, facilitating the extraction of various types of features, including first-order, shape, gray-level co-occurrence matrix, gray-level size zone matrix, gray-level run length matrix, neighbouring gray-tone difference matrix, and gray-level dependence matrix.

First, the features with ICC<0.75 were screened, and imaging histology features of different dimensions were subjected to Z-score processing, normalizing the data to mean of 0 and variance of 1. After normalising all the data, the correlation between features was calculated using the Pearson correlation coefficient. When the correlation exceeded 0.9, only one feature was retained between any two highly correlated features. Finally, the remaining features in the training dataset were filtered using the least absolute shrinkage and selection operator regression model.

A support vector machine (SVM) classification model was developed in the training cohort based on features extracted from habitat 1, habitat 2, habitat 3 and the whole tumour with five-fold cross-validation and finally validated in an external test cohort.

### 2.5 Statistical analysis

Clinical characteristics were compared using the chi-square test or Fisher’s exact test for categorical variables and the t-test or Mann–Whitney U test for continuous variables.

The predictive performance of the models for LVSI in cervical cancer was evaluated using the area under curve (AUC) of the receiver operating characteristic curve. The accuracy, sensitivity, specificity, positive predictive value, and negative predictive value were calculated. The model with the highest AUC was validated using an external test cohort. The generalisation of the model was assessed using the Delong test to compare the predictive performance of the training and test cohorts as well as the calibration curves. Ultimately, net benefit of the model’s clinical usefulness was measured using the decision curve analysis. Statistical significance was set at P< 0.05.

## 3 Results

### 3.1 Clinical characteristics

**Table 1** presents the clinical characteristics of patients with cervical cancer. A total of 300 patients from two centres, with the mean age of 51.48 ± 10.63 and 50.35 ± 9.77 years for the training and validation cohorts, respectively, were included in the study. Among them, 161 cases were classified as FIGO stage I, 105 cases as stage II, and 34 cases as stage III. Squamous cell carcinoma was present in 226 cases, adenocarcinoma in 54 cases, and adenosquamous carcinoma in 20 cases. Significant statistical differences were observed in maximum diameter, degree of cellular differentiation, CA125 levels, and FIGO stage within the training cohort. Maximum diameter, CA125 levels, and FIGO stage also demonstrated significant statistical differences within the validation cohort. Other clinical characteristics, including the difference between LVSI+ and LVSI- groups, did not show statistically significant differences in both training and external testing cohorts.

**Table 1:** Characteristics of cervical cancer patients in training and external test cohorts.

Characteristic	Training cohort			Test cohort		
	LVSI-	LVSI+	P	LVSI-	LVSI+	P
Age	51.63 ± 10.84	51.35 ± 10.49	0.853	51.81 ± 8.73	49.06 ± 10.52	0.156
Maximum diameter	22.94 ± 11.66	34.31 ± 12.75	<0.001	3.14 ± 1.45	3.85 ± 1.12	0.007
Histological type			0.161			0.717
Squamous cell carcinoma	64 (68.09)	83 (79.81)		36 (75.00)	43 (79.63)	
Adenocarcinoma	24 (25.53)	16 (15.38)		8 (16.67)	6 (11.11)	
Adenosquamous carcinoma	6 (6.38)	5 (4.81)		4 (8.33)	5 (9.26)	
Degree of cellular differentiation			<0.001			0.737
Low	10 (10.64)	21 (20.19)		11 (22.92)	16 (29.63)	
Middle	68 (72.34)	82 (78.85)		23 (47.92)	23 (42.59)	
High	16 (17.02)	1 (0.96)		14 (29.17)	15 (27.78)	
HPV			0.275			0.868
Negative	48 (51.06)	44 (42.31)		15 (31.25)	15 (27.78)	
Positive	46 (48.94)	60 (57.69)		33 (68.75)	39 (72.22)	
CA125			0.046			0.481
≤35	79 (84.04)	74 (71.15)		36 (75.00)	36 (66.67)	
>35	15 (15.96)	30 (28.85)		12 (25.00)	18 (33.33)	
CA199			0.878			0.456
≤27	75 (79.79)	81 (77.88)		37 (77.08)	37 (68.52)	
>27	19 (20.21)	23 (22.12)		11 (22.92)	17 (31.48)	
SCC			0.622			0.582
≤1.5	44 (46.81)	44 (42.31)		24 (50.00)	23 (42.59)	
>1.5	50 (53.19)	60 (57.69)		24 (50.00)	31 (57.41)	
FIGO stage			0.001			0.04
I	63 (67.02)	47 (45.19)		29 (60.42)	22 (40.74)	
II	29 (30.85)	43 (41.35)		15 (31.25)	18 (33.33)	
III	2 (2.13)	14 (13.46)		4 (8.33)	14 (25.93)	

CEA, carcinoembryonic antigen; CA125, carbohydrate antigen 125; CA19-9, carbohydrate antigen 19-9; HPV, Human papillomavirus; FIGO, International Federation of Gynecology and Obstetrics; SCC, Squamous cell carcinoma antigen.

### 3.2 Feature selection

A total of 1016 histological features were extracted from the imaging data based on habitat 1, habitat 2, habitat 3, and the whole tumour. After screening the features using ICC values<0.75, the remaining number of imaging histological features based on habitat 1, habitat 2, habitat 3, and the whole tumour were 713, 617, 692, and 627, respectively. Pearson correlation coefficients were used for filtering, resulting in 190, 148, 170, and 155 features remaining for habitat 1, habitat 2, habitat 3, and the whole tumour, respectively. The remaining imaging histological features of the training cohort were screened using the least absolute shrinkage and selection operator regression method for model building, yielding 19, 18, 19, and 7 best imaging histological features based on habitat 1, habitat 2, habitat 3, and the whole tumour, respectively. These results are presented in the **Supplementary Materials**.

### 3.3 Performance evaluation of radiomics based on habitat imaging

**Figure 3:**
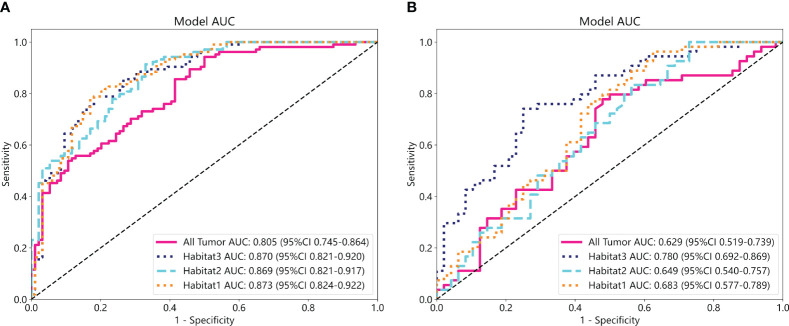
The ROC curves of the SVM machine learning models in the training **(A)** and external test cohorts **(B)**.

**Figure 4:**
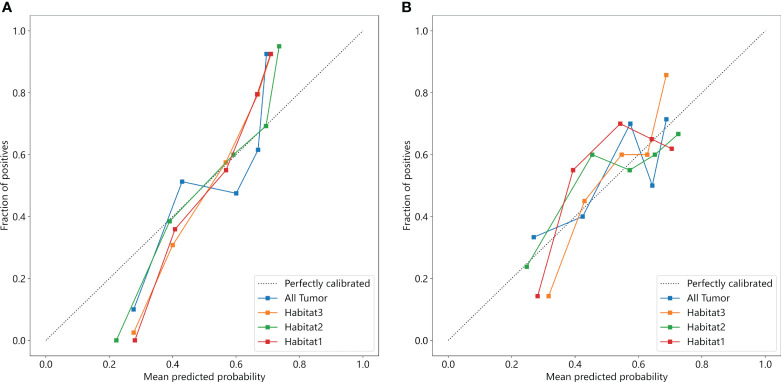
The calibration curves in the training **(A)** and external test cohorts **(B)**.

**Figure 5:**
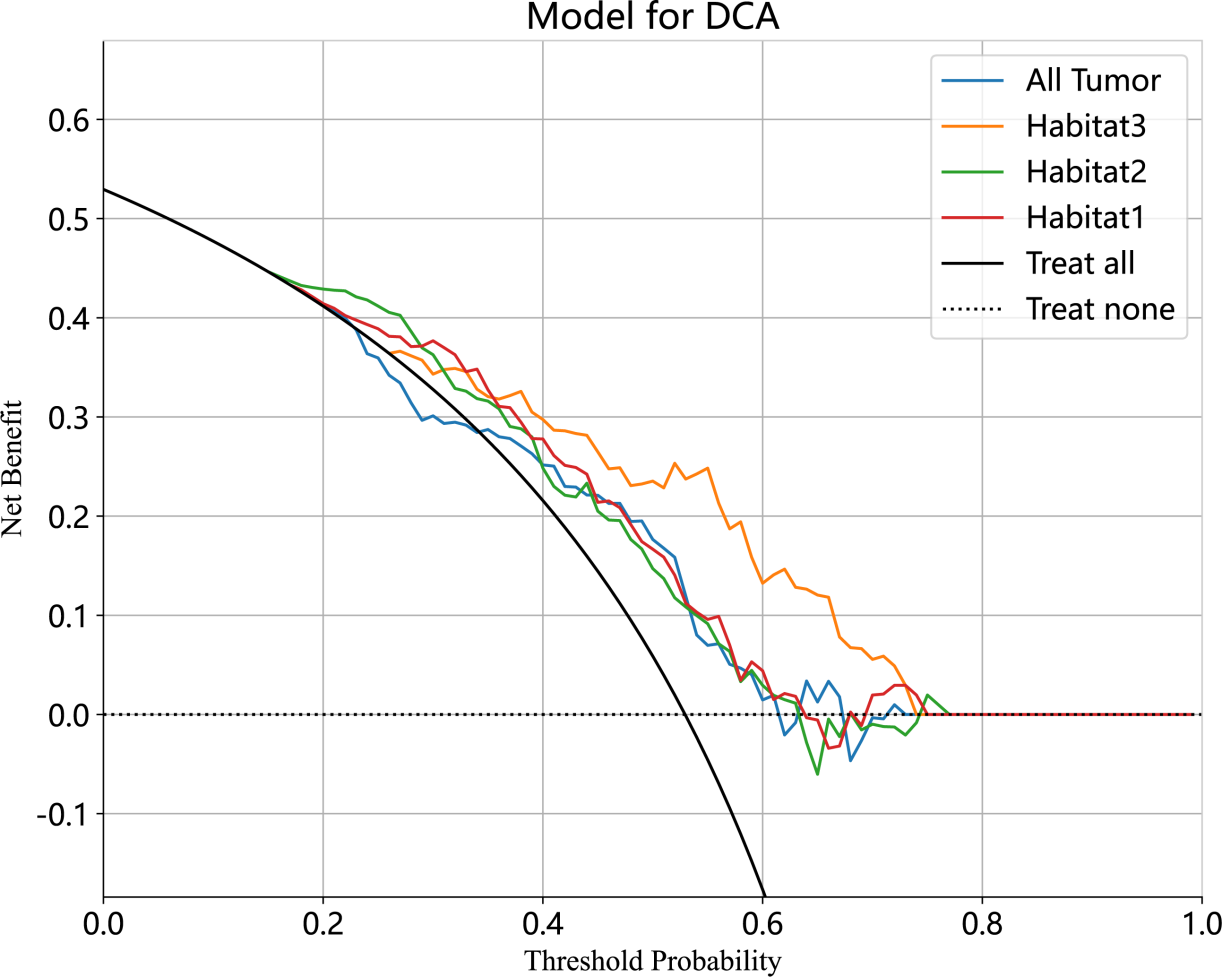
The decision curve analyses of the radiomic model in external test cohort. The Habitat3-based SVM model achieved a great net effect.

**Figure 6:**
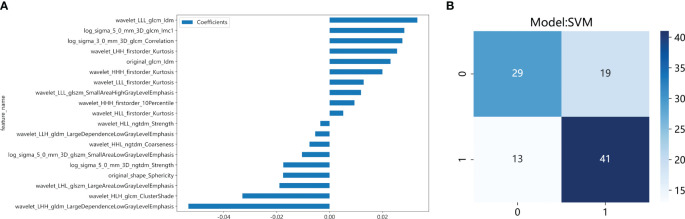
The feature weight map **(A)** and confusion matrix **(B)** of the Habitat3 radiomics model.

We developed SVM machine learning models based on the most distinctive imaging histological characteristics of habitat 1, habitat 2, habitat 3, and the whole tumour. The prediction efficiency of each model is summarized in **Table 2**. **Figure 3** illustrates the receiver operating characteristic curves of the SVM machine learning models, with area under the curves (AUCs) of 0.805 (95% confidence interval [CI]: 0.745–0.864), 0.873 (95% CI: 0.824–0.922), 0.869 (95% CI: 0.821–0.917), and 0.870 (95% CI: 0.821–0.920) for habitat 1, habitat 2, habitat 3, and the whole tumour, respectively. The external test cohort had AUCs of 0.629 (95% CI: 0.519–0.739), 0.683 (95% CI: 0.577–0.789), 0.649 (95% CI: 0.540–0.757) and 0.780 (95% CI: 0.692–0.869) for habitat 1, habitat 2, habitat 3, and the whole tumour, respectively. The habitat 3 model demonstrated superior performance than that of the whole tumour model in the external test cohort. **Figure 4** displays the calibration curves for the training and validation cohorts, showing better calibration for both groups. **Figure 5** presents the decision curve analysis curves for the external validation cohort of the model, with significant net gains observed for the habitat 3-based SVM model. Thus, the clinical importance of our model for early cervical cancer diagnosis was highlighted. **Figure 6** presents the feature weight map and confusion matrix of the habitat 3 imaging histological model. The Delong test revealed statistically significant differences between habitat 3 and the whole tumour models in both the training and validation cohorts.

**Table 2:** LVSI prediction performance of SVM model.

Models	Task	AUC	95% CI	Accuracy	Sensitivity	Specificity	PPV	NPV	P
Habitat1	train	0.873	0.824 - 0.922	0.803	0.779	0.830	0.835	0.772	0.015
	test	0.683	0.577 - 0.789	0.686	0.963	0.375	0.634	0.900	0.346
Habitat2	train	0.869	0.821 - 0.917	0.798	0.913	0.670	0.754	0.875	0.023
	test	0.649	0.540 - 0.757	0.647	0.833	0.438	0.625	0.700	0.729
Habitat3	train	0.870	0.821 - 0.920	0.803	0.788	0.819	0.828	0.778	0.018
	test	0.780	0.692 - 0.869	0.745	0.741	0.750	0.769	0.720	0.006
Whole tumour	train	0.805	0.745 - 0.864	0.732	0.942	0.500	0.676	0.887	ref
	test	0.629	0.519 - 0.739	0.657	0.778	0.521	0.646	0.676	ref

AUC, area under the curve; PPV, positive predictive value; NPV, negative predictive value.

P values are derived from the DeLong’s test of AUCs where AUC of whole tumour is the reference standard for comparison.

## 4 Discussion

In this study, three sub-regions were delineated based on voxel and entropy values from contrast-enhanced T1-weighted imaging (CE-T1WI) of cervical cancer using habitat analysis, which is a heterogeneous metric. The SVM models based on the three habitat sub-regions exhibited a higher predictive performance for LVSI in cervical cancer than those derived from the whole tumour. Notably, the highest AUC of 0.870 (95% CI: 0.821–0.920) was derived from habitat 3, and this performance was robust across different centres (the AUC of the model in the external test cohort was 0.780 (95% CI: 0.692–0.869), and the difference between the training and external test cohorts was not statistical significant. The performance of the models in predicting LVSI was compared, and we observed that the prediction models built based on habitat 3 outperformed conventional overall tumour model in the training and external test cohorts with an AUC of 0.780 (95% CI: 0.692–0.869). This indicated that the tumour sub-regional radiomics model based on habitat analysis could enhance LVSI prediction in cervical cancer.

Cervical cancer primarily metastasizes through blood or lymphatic vessels to other body tissues (22). Previous studies have indicated that the presence of LVSI implies a higher risk of lymph node metastasis and a greater probability of lymph node micrometastasis when LVSI is positive (23). LVSI is widely recognised as a risk factor for cervical cancer and directly affects the prognosis of patients with cervical cancer (24). The treatment of cervical cancer varies according to the stage and the presence of LVSI in patients with clinical stage IA (8). In the absence of LVSI, cervical conization alone is necessary to avoid radical hysterectomy. Therefore, the preoperative evaluation of LVSI is essential (25–27).

In the final analysis, we included 300 patients. In the training and validation cohorts, the difference between FIGO staging and LVSI status was statistically significant. The probability of LVSI occurrence increased from 42.86% (69/161) in stage IB to 58.1% (61/105) and 82.35% (28/34) in stages II and III, respectively, suggesting a greater that the probability of LVSI occurrence increased progressively with the advancing stage of cervical cancer. In our study, squamous cell carcinoma was present in 226, adenocarcinoma in 54, and adenosquamous carcinoma in 20 patients. The difference between the histological type and LVSI in the training and validation cohorts was not statistically significant, thus indicating that the histological type of cervical cancer did not affect the occurrence of LVSI in patients with cervical cancer (28).

Compared to whole-tumour radiomics, habitat imaging, an approach focused on sub-region imaging omics analysis, offers better quantification of tumour sub-regions that are more relevant to tumour growth or invasiveness (15). Invasive sub-regions have been reported to be important for prognosis and treatment response (29, 30). Fang et al. utilised a variety of tumour habitat features within a radiomics model to predict treatment responses in patients with locally advanced cervical cancer before synchronous chemoradiotherapy (31). The model, consisting of three habitat features derived from multi-parametric MR images, demonstrated good predictive performance with AUCs of 0.820 and 0.798 in the training and test sets, respectively, outperforming a single MR parameter model. Cho et al. derived habitat images from dynamic contrast-enhanced magnetic resonance imaging of breast cancer and extracted radiomic features to establish a breast cancer habitat risk score that could accurately categorise patients into high-risk and low-risk groups (32). Choi et al. used multi-parametric MR to extract radiomic features from multiple habitats of the tumour and identified three different subtypes through consistent clustering, revealing different phenotypic subtypes of glioblastoma with clinical and genomic significance. This approach highlights the potential of radiomics as a prognostic biomarker by using multi-habitat imaging (33).

In this study, we employed CE-T1WI images to conduct a clustering analysis, enabling the effective evaluation of blood perfusion in the body by displaying vascular density and perfusion. Additionally, we measured the volume transfer constant, which relied on the permeability of tumour blood vessels (34). This approach provided more discriminatory information for predicting LVSI invasion in cervical cancer. Our prediction results indicated that the radiomics model based on habitat3 outperformed the whole tumour in both the training and external validation sets. The heterogeneous nature of solid tumours suggested that LVSI in cervical cancer might not be distributed uniformly and could exhibit variations at the microscale voxel level. After clustering the image voxels and entropy values, habitat3 was observed to contain more LVSI information, whereas the whole tumour comprised complete heterogeneous information. Our utilisation of habitat, a novel technology for clustering solid tumours in preoperative imaging and subsequently extracting radiomic features from the clustered tumour sub-regions, helped to avoid the inclusion of irrelevant areas that are not related to LVSI in cervical cancer in the feature extraction process, thereby improving the model’s predictive performance.

This study had some limitations. First, although this study included a larger number of patients than that of previous studies, a larger prospective dataset will be required to further improve the model’s performance. Second, the diversity in the settings of multi-centre MR devices could have introduced variability in MR images due to differences in equipment and scanning parameters. Thus, we made efforts to standardise and normalise the images as much as possible to eliminate the effect of equipment-related differences.

In conclusion, the sub-region-based approach could predict the LVSI status in cervical cancer demonstrating superior performance over traditional radiomics of the whole tumour, thus making it a promising non-invasive biomarker for predicting preoperative LVSI, especially in patients with cervical cancer. The external test cohort demonstrated the model’s stable performance with a strong AUC.

## Data availability statement

The raw data supporting the conclusions of this article will be made available by the authors, without undue reservation.

## Ethics statement

The studies involving humans were approved by The Medical Ethical Committee of Shenzhen People’s Hospital and Guangzhou Women and Children's Medical Center. The studies were conducted in accordance with the local legislation and institutional requirements. The participants provided their written informed consent to participate in this study.

## Author contributions

JG: Conceptualization, Writing-review and editing; SW and XL: Data curation, Formal analysis, Resources, Software and Writing-original draft; XL and YW: Investigation; CJ and YL: Methodology and Validation; XT: Project administration; RW and XZ: Supervision. All authors contributed to the article and approved the submitted version.

## References

[1] Miller KD Nogueira L Devasia T Mariotto AB Yabroff KR Jemal A. Cancer treatment and survivorship statistics, 2022. CA Cancer J Clin (2022) 72:409–36. doi: 10.3322/caac.21731 35736631

[2] Sung H Ferlay J Siegel RL Laversanne M Soerjomataram I Jemal A. Global cancer statistics 2020: GLOBOCAN estimates of incidence and mortality worldwide for 36 cancers in 185 countries. CA Cancer J Clin (2021) 71:209–49. doi: 10.3322/caac.21660 33538338

[3] Small W Bacon MA Bajaj A Chuang LT Fisher BJ Harkenrider MM. Cervical cancer: A global health crisis. Cancer (2017) 123:2404–12. doi: 10.1002/cncr.30667 28464289

[4] Ferlay J Colombet M Soerjomataram I Mathers C Parkin DM Piñeros M. Estimating the global cancer incidence and mortality in 2018: GLOBOCAN sources and methods. Int J Cancer (2019) 144:1941–53. doi: 10.1002/ijc.31937 30350310

[5] Margolis B Cagle-Colon K Chen L Tergas AI Boyd L Wright JD. Prognostic significance of lymphovascular space invasion for stage IA1 and IA2 cervical cancer. Int J Gynecol Cancer (2020) 30:735–43. doi: 10.1136/ijgc-2019-000849 32179697

[6] Shirabe K Itoh S Yoshizumi T Soejima Y Taketomi A Aishima S-I. The predictors of microvascular invasion in candidates for liver transplantation with hepatocellular carcinoma-with special reference to the serum levels of des-gamma-carboxy prothrombin. J Surg Oncol (2007) 95:235–40. doi: 10.1002/jso.20655 17323337

[7] Yoneda JY Braganca JF Sarian LO Borba PP Conceição JCJ Zeferino LC. Surgical treatment of microinvasive cervical cancer: analysis of pathologic features with implications on radicality. Int J Gynecol Cancer (2015) 25:694–8. doi: 10.1097/IGC.0000000000000416 25742569

[8] Bhatla N Aoki D Sharma DN Sankaranarayanan R. Cancer of the cervix uteri. Int J Gynaecol Obstet (2018) 143(Suppl 2):22–36. doi: 10.1002/ijgo.12611 30306584

[9] Gillies RJ Brown JS Anderson ARA Gatenby RA. Eco-evolutionary causes and consequences of temporal changes in intratumoural blood flow. Nat Rev Cancer (2018) 18:576–85. doi: 10.1038/s41568-018-0030-7 PMC6441333 29891961

[10] Junttila MR de Sauvage FJ. Influence of tumour micro-environment heterogeneity on therapeutic response. Nature (2013) 501:346–54. doi: 10.1038/nature12626 24048067

[11] Janiszewska M. The microcosmos of intratumor heterogeneity: the space-time of cancer evolution. Oncogene (2020) 39:2031–9. doi: 10.1038/s41388-019-1127-5 PMC7374939 31784650

[12] Gatenby RA Grove O Gillies RJ. Quantitative imaging in cancer evolution and ecology. Radiology (2013) 269:8–15. doi: 10.1148/radiol.13122697 24062559 PMC3781355

[13] Dextraze K Saha A Kim D Narang S Lehrer M Rao A. Spatial habitats from multiparametric MR imaging are associated with signaling pathway activities and survival in glioblastoma. Oncotarget (2017) 8:112992–3001. doi: 10.18632/oncotarget.22947 PMC5762568 29348883

[14] Kim J Ryu S-Y Lee S-H Lee HY Park H. Clustering approach to identify intratumour heterogeneity combining FDG PET and diffusion-weighted MRI in lung adenocarcinoma. Eur Radiol (2019) 29:468–75. doi: 10.1007/s00330-018-5590-0 29922931

[15] Wu J Cao G Sun X Lee J Rubin DL Napel S. Intratumoral spatial heterogeneity at perfusion MR imaging predicts recurrence-free survival in locally advanced breast cancer treated with neoadjuvant chemotherapy. Radiology (2018) 288:26–35. doi: 10.1148/radiol.2018172462 29714680 PMC6029132

[16] Park JE Kim HS Kim N Park SY Kim Y-H Kim JH. Spatiotemporal heterogeneity in multiparametric physiologic MRI is associated with patient outcomes in IDH-wildtype glioblastoma. Clin Cancer Res (2021) 27:237–45. doi: 10.1158/1078-0432.CCR-20-2156 33028594

[17] Gillies RJ Kinahan PE Hricak H. Radiomics: images are more than pictures, they are data. Radiology (2016) 278:563–77. doi: 10.1148/radiol.2015151169 PMC4734157 26579733

[18] Xie C Yang P Zhang X Xu L Wang X Li X. Sub-region based radiomics analysis for survival prediction in oesophageal tumours treated by definitive concurrent chemoradiotherapy. EBioMedicine (2019) 44:289–97. doi: 10.1016/j.ebiom.2019.05.023 PMC6606893 31129097

[19] Chen L Liu K Zhao X Shen H Zhao K Zhu W. Habitat imaging-based 18F-FDG PET/CT radiomics for the preoperative discrimination of non-small cell lung cancer and benign inflammatory diseases. Front Oncol (2021) 11:759897. doi: 10.3389/fonc.2021.759897 34692548 PMC8526895

[20] van Griethuysen JJM Fedorov A Parmar C Hosny A Aucoin N Narayan V. Computational radiomics system to decode the radiographic phenotype. Cancer Res (2017) 77:e104–7. doi: 10.1158/0008-5472.CAN-17-0339 PMC5672828 29092951

[21] Zwanenburg A Vallières M Abdalah MA Aerts HJWL Andrearczyk V Apte A. The image biomarker standardization initiative: standardized quantitative radiomics for high-throughput image-based phenotyping. Radiology (2020) 295:328–38. doi: 10.1148/radiol.2020191145 PMC7193906 32154773

[22] Agarwal U Dahiya P Chauhan A Sangwan K Purwar P. Scalp metastasis in carcinoma of the uterine cervix–a rare entity. Gynecol Oncol (2002) 87:310–2. doi: 10.1006/gyno.2002.6829 12468331

[23] Li K Sun H Lu Z Xin J Zhang L Guo Y. Value of [18F]FDG PET radiomic features and VEGF expression in predicting pelvic lymphatic metastasis and their potential relationship in early-stage cervical squamous cell carcinoma. Eur J Radiol (2018) 106:160–6. doi: 10.1016/j.ejrad.2018.07.024 30150039

[24] Parra-Herran C Taljaard M Djordjevic B Reyes MC Schwartz L Schoolmeester JK. Pattern-based classification of invasive endocervical adenocarcinoma, depth of invasion measurement and distinction from adenocarcinoma in situ: interobserver variation among gynecologic pathologists. Mod Pathol (2016) 29:879–92. doi: 10.1038/modpathol.2016.86 27174588

[25] Fang J Zhang B Wang S Jin Y Wang F Ding Y. Association of MRI-derived radiomic biomarker with disease-free survival in patients with early-stage cervical cancer. Theranostics (2020) 10:2284–92. doi: 10.7150/thno.37429 PMC7019161 32089742

[26] Luo Y Mei D Gong J Zuo M Guo X. Multiparametric MRI-based radiomics nomogram for predicting lymphovascular space invasion in endometrial carcinoma. J Magn Reson Imaging (2020) 52:1257–62. doi: 10.1002/jmri.27142 32315482

[27] Mazzola R Ricchetti F Fiorentino A Levra NG Fersino S Di Paola G. Weekly cisplatin and volumetric-modulated arc therapy with simultaneous integrated boost for radical treatment of advanced cervical cancer in elderly patients: feasibility and clinical preliminary results. Technol Cancer Res Treat (2017) 16(3):310–5. doi: 10.1177/1533034616655055 PMC5616045 27402633

[28] Arezzo F Cormio G Mongelli M Cazzato G Silvestris E Kardhashi A. Machine learning applied to MRI evaluation for the detection of lymph node metastasis in patients with locally advanced cervical cancer treated with neoadjuvant chemotherapy. Arch Gynecol Obstet (2023) 307(6):1911–9. doi: 10.1007/s00404-022-06824-6 36370209

[29] Cui Y Tha KK Terasaka S Yamaguchi S Wang J Kudo K. Prognostic imaging biomarkers in glioblastoma: development and independent validation on the basis of multiregion and quantitative analysis of MR images. Radiology (2016) 278:546–53. doi: 10.1148/radiol.2015150358 PMC4734164 26348233

[30] Zhou M Chaudhury B Hall LO Goldgof DB Gillies RJ Gatenby RA. Identifying spatial imaging biomarkers of glioblastoma multiforme for survival group prediction. J Magn Reson Imaging (2017) 46:115–23. doi: 10.1002/jmri.25497 27678245

[31] Fang M Kan Y Dong D Yu T Zhao N Jiang W. Multi-habitat based radiomics for the prediction of treatment response to concurrent chemotherapy and radiation therapy in locally advanced cervical cancer. Front Oncol (2020) 10:563. doi: 10.3389/fonc.2020.00563 32432035 PMC7214615

[32] Cho H Kim H Nam SY Lee JE Han B-K Ko EY. Measurement of perfusion heterogeneity within tumor habitats on magnetic resonance imaging and its association with prognosis in breast cancer patients. Cancers (2022) 14:1858. doi: 10.3390/cancers14081858 35454768 PMC9025287

[33] Choi SW Cho H-H Koo H Cho KR Nenning K-H Langs G. Multi-habitat radiomics unravels distinct phenotypic subtypes of glioblastoma with clinical and genomic significance. Cancers (2020) 12:1707. doi: 10.3390/cancers12071707 32605068 PMC7408408

[34] Ellingsen C Walenta S Hompland T Mueller-Klieser W Rofstad EK. The microenvironment of cervical carcinoma xenografts: associations with lymph node metastasis and its assessment by DCE-MRI. Transl Oncol (2013) 6:607–17. doi: 10.1593/tlo.13313 PMC3799202 24151541

