# Supplementary material for: Habitat-based radiomics enhances the ability to predict lymphovascular space invasion in cervical cancer: a multi-center study
Source: Front Oncol. 2023 Oct 26;13:1252074. doi: 10.3389/fonc.2023.1252074 (PMC10637586; doi:10.3389/fonc.2023.1252074)
Supplement: Supplementary file 1 [file DataSheet_1.docx]

**Supplementary Material**

**Supplementary Methods 1.** Magnetic resonance image acquisition parameters used in the present study.

| MR Scanner | repetition time / echo time (TR/TE ms) | field-of-view (FOV mm) | slice thickness (mm) |
| --- | --- | --- | --- |
| 3.0T MAGENTO Prisma (Siemens) | 4.21/2.04 | 320×320 | 3 |
| 3.0T Magnetom Skyra (Siemens) | 4.03/1.97 | 330×330 | 3 |
| 1.5T uMR 660 (United Imaging) | 4.78/2.23 | 350×350 | 5 |
| 1.5T Magneton Avanto (Siemens) | 5.77/2.66 | 320×320 | 2.5 |

Contrast agent was injected intravenously at a rate of 2ml/s, the injection dose was 0.2mmol/kg, and then a bolus of 20ml of 0.9% NaCl solution was performed in both institutions. The MR scan covers the area from the superior border of the iliac crest to the inferior border of the pubic symphysis.

**Supplementary Methods 2.**

**Table 2.1** Selected features and weight coefficient

| Models | Features | Weight coefficient |
| --- | --- | --- |
| All Tumor | log_sigma_2_0_mm_3D_glcm_Imc2 | -0.001768 |
|  | log_sigma_2_0_mm_3D_ngtdm_Busyness | 0.008916 |
|  | log_sigma_2_0_mm_3D_ngtdm_Strength | -0.014897 |
|  | log_sigma_3_0_mm_3D_glcm_Idn | 0.028748 |
|  | wavelet_HLL_glszm_LargeAreaLowGrayLevelEmphasis | -0.004411 |
|  | wavelet_LHH_firstorder_Kurtosis | 0.02411 |
|  | wavelet_LHL_ngtdm_Coarseness | -0.054419 |
| Habitat1 | log_sigma_2_0_mm_3D_firstorder_RootMeanSquared | -0.024472 |
|  | log_sigma_2_0_mm_3D_glcm_Imc2 | -0.031317 |
|  | log_sigma_3_0_mm_3D_glcm_Imc2 | -0.002486 |
|  | log_sigma_3_0_mm_3D_gldm_DependenceVariance | 0.024561 |
|  | log_sigma_3_0_mm_3D_ngtdm_Coarseness | -0.025254 |
|  | log_sigma_5_0_mm_3D_glcm_Imc2 | -0.037894 |
|  | log_sigma_5_0_mm_3D_ngtdm_Strength | -0.000402 |
|  | original_firstorder_Kurtosis | 0.031205 |
|  | original_firstorder_Range | 0.006645 |
|  | original_glszm_HighGrayLevelZoneEmphasis | 0.003479 |
|  | wavelet_HHH_glszm_SmallAreaLowGrayLevelEmphasis | -0.016323 |
|  | wavelet_HHL_firstorder_90Percentile | -0.013708 |
|  | wavelet_HHL_glcm_ClusterShade | -0.031972 |
|  | wavelet_HHL_glszm_SmallAreaHighGrayLevelEmphasis | 0.050073 |
|  | wavelet_HLL_glcm_DifferenceAverage | -0.022723 |
|  | wavelet_HLL_glcm_DifferenceVariance | -0.019641 |
|  | wavelet_LHH_gldm_LargeDependenceLowGrayLevelEmphasis | -0.038663 |
|  | wavelet_LHL_firstorder_RootMeanSquared | -0.02741 |
|  | wavelet_LLL_glcm_Idn | 0.03821 |
| Habitat2 | log_sigma_2_0_mm_3D_ngtdm_Strength | -0.009179 |
|  | log_sigma_3_0_mm_3D_glcm_Idm | 0.058267 |
|  | log_sigma_5_0_mm_3D_glcm_Imc1 | 0.029577 |
|  | original_gldm_LargeDependenceLowGrayLevelEmphasis | -0.018583 |
|  | original_glszm_LargeAreaLowGrayLevelEmphasis | -0.01534 |
|  | wavelet_HHL_firstorder_10Percentile | 0.021536 |
|  | wavelet_HHL_glcm_Idn | 0.00563 |
|  | wavelet_HHL_gldm_LargeDependenceHighGrayLevelEmphasis | 0.009625 |
|  | wavelet_HHL_glszm_HighGrayLevelZoneEmphasis | 0.016133 |
|  | wavelet_HLH_firstorder_RootMeanSquared | -0.048627 |
|  | wavelet_HLH_gldm_LargeDependenceLowGrayLevelEmphasis | -0.026938 |
|  | wavelet_HLL_glcm_Correlation | 0.018484 |
|  | wavelet_LHH_firstorder_Kurtosis | 0.037433 |
|  | wavelet_LHH_glszm_SmallAreaLowGrayLevelEmphasis | -0.008593 |
|  | wavelet_LHH_ngtdm_Coarseness | -0.02559 |
|  | wavelet_LHL_firstorder_Kurtosis | 0.002841 |
|  | wavelet_LLL_glcm_InverseVariance | 0.007384 |
|  | wavelet_LLL_ngtdm_Complexity | 0.006453 |
| Habitat3 | log_sigma_3_0_mm_3D_glcm_Correlation | 0.027521 |
|  | log_sigma_5_0_mm_3D_glcm_Imc1 | 0.028233 |
|  | log_sigma_5_0_mm_3D_glszm_SmallAreaLowGrayLevelEmphasis | -0.010446 |
|  | log_sigma_5_0_mm_3D_ngtdm_Strength | -0.017507 |
|  | original_glcm_Idm | 0.023016 |
|  | original_shape_Sphericity | -0.017593 |
|  | wavelet_HHH_firstorder_10Percentile | 0.009404 |
|  | wavelet_HHH_firstorder_Kurtosis | 0.019965 |
|  | wavelet_HHL_ngtdm_Coarseness | -0.007589 |
|  | wavelet_HLH_glcm_ClusterShade | -0.032964 |
|  | wavelet_HLL_firstorder_Kurtosis | 0.005156 |
|  | wavelet_HLL_ngtdm_Strength | -0.003508 |
|  | wavelet_LHH_firstorder_Kurtosis | 0.025502 |
|  | wavelet_LHH_gldm_LargeDependenceLowGrayLevelEmphasis | -0.053368 |
|  | wavelet_LHL_glszm_LargeAreaLowGrayLevelEmphasis | -0.018978 |
|  | wavelet_LLH_gldm_LargeDependenceLowGrayLevelEmphasis | -0.005468 |
|  | wavelet_LLL_firstorder_Kurtosis | 0.01288 |
|  | wavelet_LLL_glcm_Idm | 0.033183 |
|  | wavelet_LLL_glszm_SmallAreaHighGrayLevelEmphasis | 0.01188 |


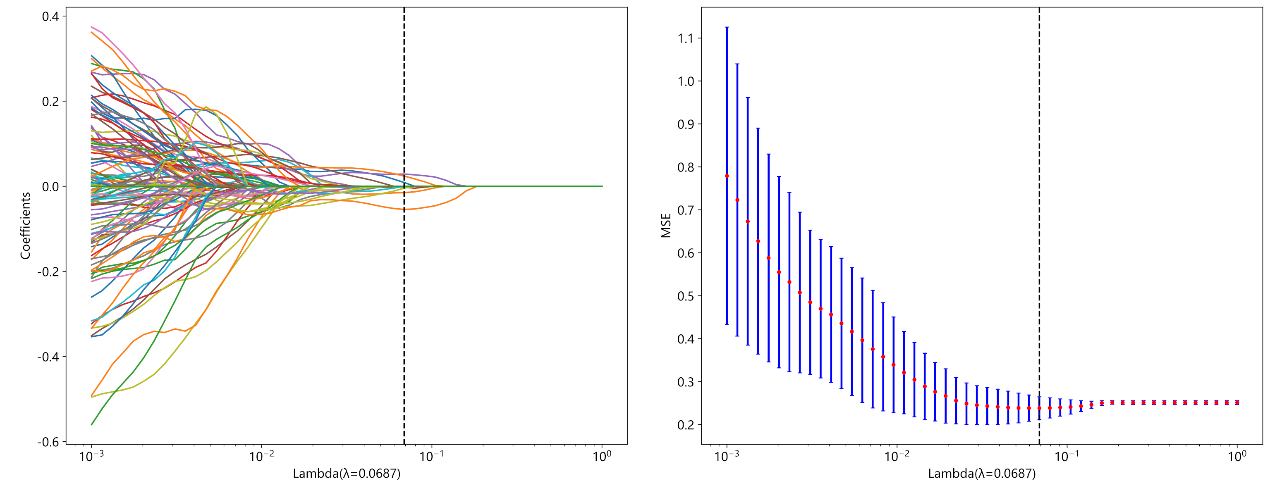


**Figure 2.1** Based on all tumor radiomics features, the best radiomics features were filtered by LASOO regression. A. Selection of the best radiomics features with non-zero coefficients. B. The LASSO model selects the optimal parameter (λ).


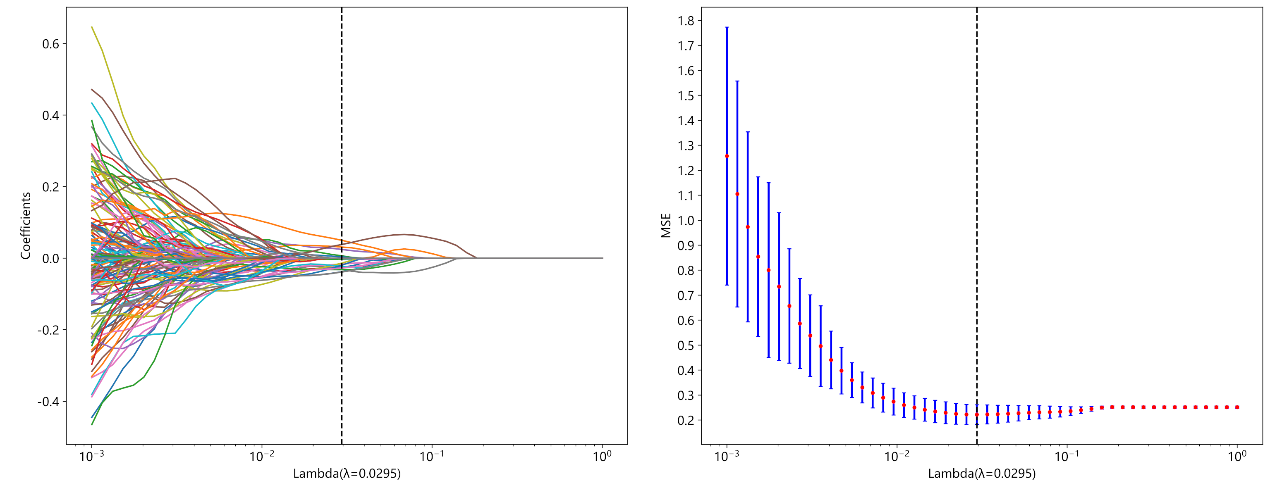


**Figure 2.2** Based on Habitat1 radiomics features, the best radiomics features were filtered by LASOO regression. A. Selection of the best radiomics features with non-zero coefficients. B. The LASSO model selects the optimal parameter (λ).


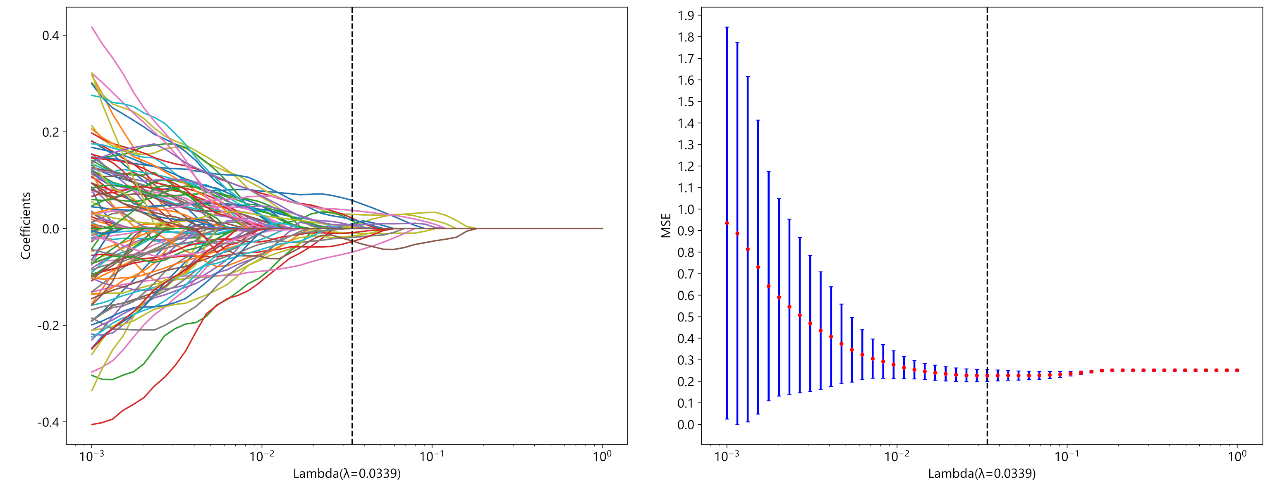


**Figure 2.3** Based on Habitat2 radiomics features, the best radiomics features were filtered by LASOO regression. A. Selection of the best radiomics features with non-zero coefficients. B. The LASSO model selects the optimal parameter (λ).


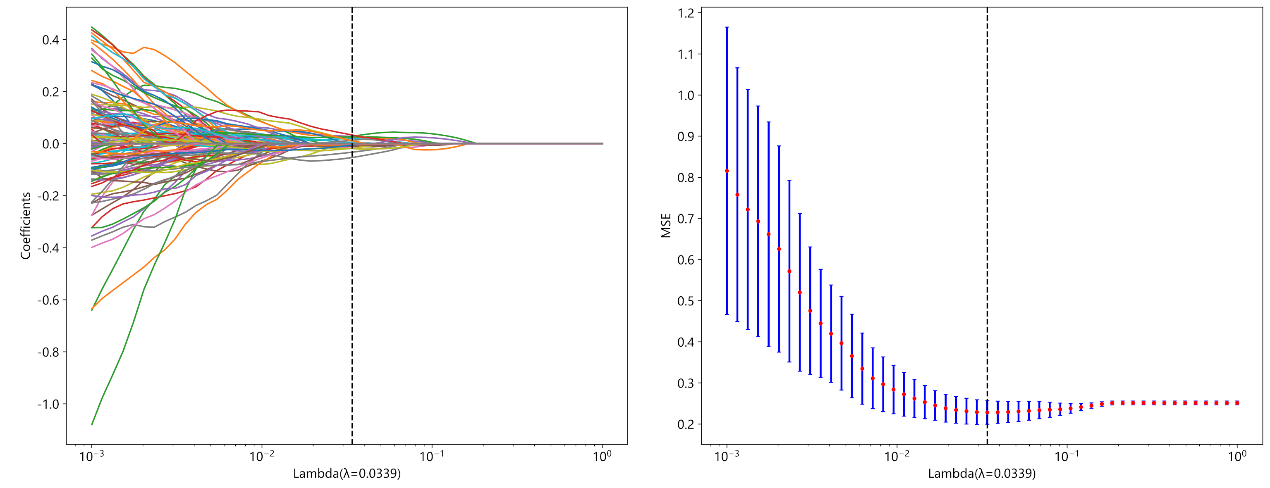


**Figure 2.4** Based on Habitat3 radiomics features, the best radiomics features were filtered by LASOO regression. A. Selection of the best radiomics features with non-zero coefficients. B. The LASSO model selects the optimal parameter (λ).
